# Unusual Metastatic Patterns of Invasive Lobular Carcinoma of the Breast

**DOI:** 10.1155/2013/986517

**Published:** 2013-11-10

**Authors:** Justin D. Sobinsky, Thomas D. Willson, Francis J. Podbielski, Mark M. Connolly

**Affiliations:** St. Joseph Hospital, Department of Surgery, 2900 North Lake Shore Drive, Chicago, IL 60657, USA

## Abstract

Invasive lobular carcinoma of the breast has similar patterns of metastatic disease when compared to invasive ductal carcinoma; however, lobular carcinoma metastasizes to unusual sites more frequently. We present a 65-year-old female with a history of invasive lobular breast carcinoma (T3N3M0) treated with modified radical mastectomy and aromatase-inhibitor therapy who underwent a surveillance PET scan, which showed possible sigmoid cancer. Colonoscopy with biopsy revealed a 3 cm sigmoid adenocarcinoma. The patient underwent a lower anterior resection. Pathology showed an ulcerated, invasive moderately differentiated adenocarcinoma extending into but not through the muscularis propria. However, six of seventeen paracolonic lymph nodes were positive for metastatic breast carcinoma (ER+/PR+), consistent with her lobular primary breast carcinoma; there was no evidence of metastatic colon cancer. This case highlights the unusual metastatic patterns of lobular carcinoma.

## 1. Case and Surgical Treatment

A 65-year-old female with a history of invasive lobular breast carcinoma (T3N3M0) treated with modified radical mastectomy and aromatase-inhibitor therapy underwent a surveillance PET scan approximately three years later, which showed possible sigmoid cancer (Figure 4). The patient was referred to gastroenterology; a colonoscopy with biopsy revealed a 3 cm sigmoid adenocarcinoma. 

**Figure 1:**
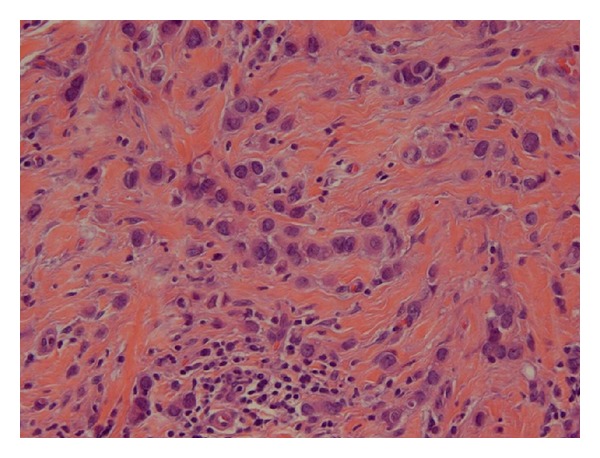
Primary lobular carcinoma.

**Figure 2:**
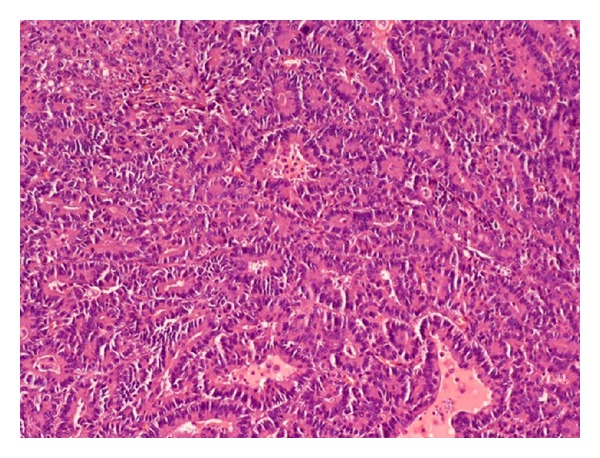
Primary colon adenocarcinoma.

**Figure 3:**
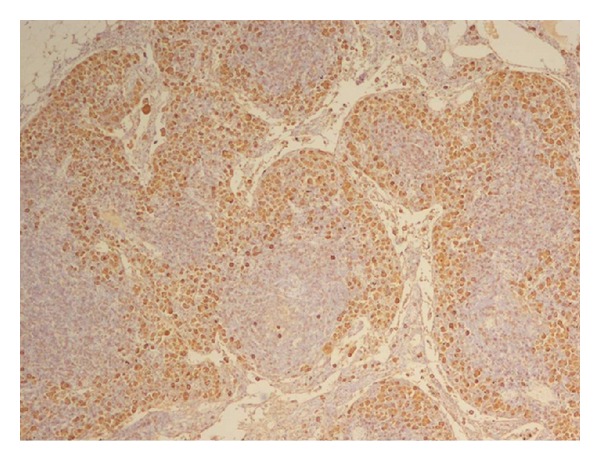
Pericolonic lymph node with positive mammaglobin staining.

**Figure 4:**
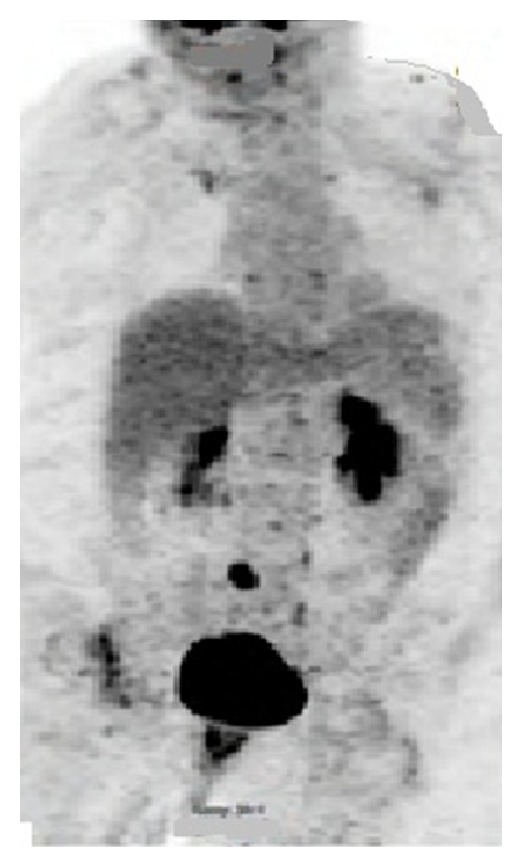
PET CT scan with avid FDG uptake associated with the sigmoid colon.

The patient underwent a lower anterior resection. Pathology showed an ulcerated, invasive moderately differentiated adenocarcinoma extending into but not through the muscularis propria (Figure 2). However, six of seventeen paracolonic lymph nodes were positive for metastatic breast carcinoma (ER+/PR+), consistent with her lobular primary breast carcinoma (Figure 1); there was no evidence of metastatic colon cancer (Figure 3). 

Hematology/oncology was consulted regarding her metastatic invasive lobular breast carcinoma. They discontinued her tamoxifen and started her on Arimidex. The patient had a recent PET scan, which showed no signs of recurrent disease.

## 2. Discussion

One in twelve American women develop breast cancer, and infiltrating lobular carcinoma (ILC) involves around 10% of these cases [1]. When comparing ILC to infiltrating ductal carcinoma (IDC), the sites of metastatic spread differ. In IDC, the common sites of metastatic disease are seen in the lung, bones, and liver. However, in ILC metastatic disease has been reported in the GI tract, peritoneum, and retroperitoneum.

For instance, Ferlicot et al. showed more diverse patterns of tumor spread in ILC when compared to IDC. There was a statistically significant difference in metastatic spread in the bones, lung, and abdominal organs. In ILC, metastatic spread was seen more frequently in the bones and abdominal region whereas IDC metastasized to the lung. There was no difference in liver, nonaxillary lymph nodes, or the central nervous system [2]. 

Other studies have shown similar findings especially in postmortem examinations. Metastatic ILC was seen more frequently in the ovaries, uterus, peritoneum, retroperitoneum, stomach, and intestine on autopsy [3]. However, metastatic spread to the lungs was more common in IDC when compared to ILC, which has been seen in previous studies [4]. Despite the fact that there is a wide range of dissemination in ILC, it has been shown that there is no difference in overall survival when compared to IDC [5, 6]. There has also been no significant difference in disease-free survival between the two [6].

It is not common to see colonic involvement in patients with a history of ILC; as high as 12% has been cited in the literature [7]. In our case the patient had evidence of metastatic lobular carcinoma in the colonic lymph nodes. Even though a patient that has been disease-free for many years, it is important to not rule out metastatic ILC in patients with symptoms or presentation of abdominal complaints. It has been reported that gastrointestinal metastases have been seen on an average of 9.5 years and possibly as late as 20 years after initial diagnosis [8]. Specifically with our patient, she had a disease-free interval of three years, well within the average. 

## 3. Conclusion 

It is important that clinicians be cognizant that ILC has a much wider and different pattern of metastatic disease when compared to IDC. Metastatic ILC must be in the differential diagnosis in a patient with a history of ILC presenting with abdominal complaints or an incidental finding seen on the screening imaging, even if the patient has been disease-free for several years.

## References

[1] Mulholland MW, Lillemoe KD, Doherty GM (2011). *Greenfield's Surgery: Scientific Principles and Practice*.

[2] Ferlicot S, Vincent-Salomon A, Médioni J (2004). Wide metastatic spreading in infiltrating lobular carcinoma of the breast. *European Journal of Cancer*.

[3] Harris M, Howell A, Chrissohou M (1984). A comparison of the metastatic pattern of infiltrating lobular carcinoma and infiltrating duct carcinoma of the breast. *British Journal of Cancer*.

[4] Lamovec J, Bracko M (1991). Metastatic pattern of infiltrating lobular carcinoma of the breast: an autopsy study. *Journal of Surgical Oncology*.

[5] Rozan S, Vincent-Salomon A, Zafrani B (1998). No significant predictive value of c-erbB-2 or p-53 expression regarding sensitivity to primary chemotherapy or radiotherapy in breast cancer. *International Journal of Cancer*.

[6] Fortunato L, Mascaro A, Poccia I (2012). Lobular breast cancer: same survival and local control compared with ductal cancer, but should both be treated the same way? Analysis of an institutional database over a 10-year period. *Annals of Surgical Oncology*.

[7] Cifuentes N, Pickren JW (1979). Metastases from carcinoma of mammary gland: an autopsy study. *Journal of Surgical Oncology*.

[8] Nazareno J, Taves D, Preiksaitis HG (2006). Metastatic breast cancer to the gastrointestinal tract: a case series and review of the literature. *World Journal of Gastroenterology*.

